# Supplementary material for: Birds multiplex spectral and temporal visual information via retinal On- and Off-channels
Source: Nat Commun. 2023 Aug 31;14:5308. doi: 10.1038/s41467-023-41032-z (PMC10471707; doi:10.1038/s41467-023-41032-z)
Supplement: Supplementary file 1 — Supplementary Information [file 41467_2023_41032_MOESM1_ESM.pdf]

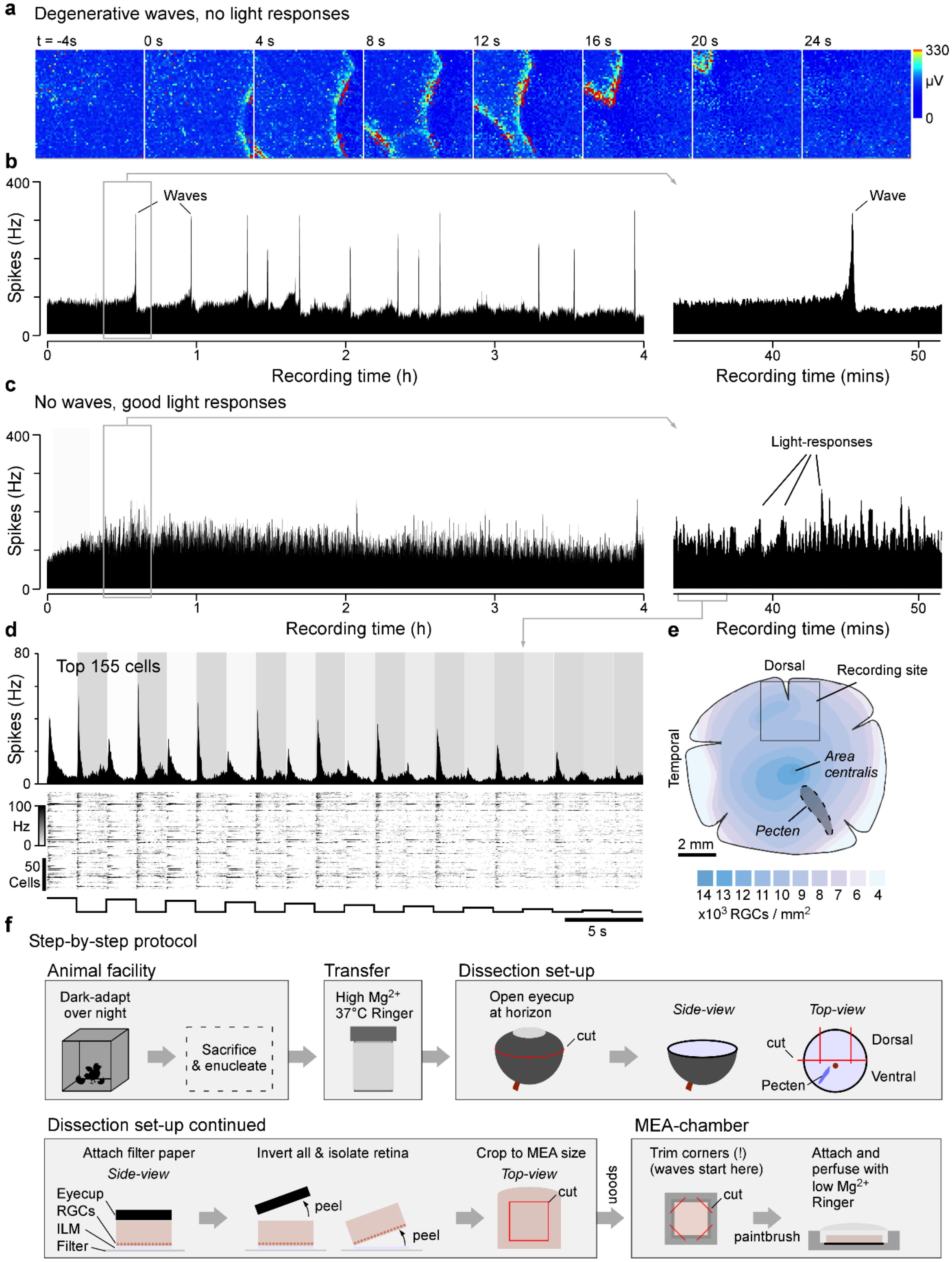

Supplemental Figure S1 – related to Figure 1. a,b, Illustrative example of ‘unhealthy’ chick retina showing spontaneous pathological waves of depression, including an example time-series of MEA frames (a), and the corresponding histogram of detected spikes across the entire array over time (b). No light-responses were

detectable in this retina. **c**, Example recording from ‘healthy’ retina yielding stable light-responses for at least four hours. **d**, Zoom in to the top 155 responding cells from (c), showing reliable and diverse light responses to the 100-10% contrast ‘white’ step stimulus (Methods). **e**, Schematic representation of the flat-mounted chicken retina with key structures, recording area and RGC densities indicated – modified from Ref[20]. **f**, Illustrative step-by-step protocol of our tissue-preparation strategy (see Methods for narrative details). Chick schematic in (f) from silhouettegarden.com.

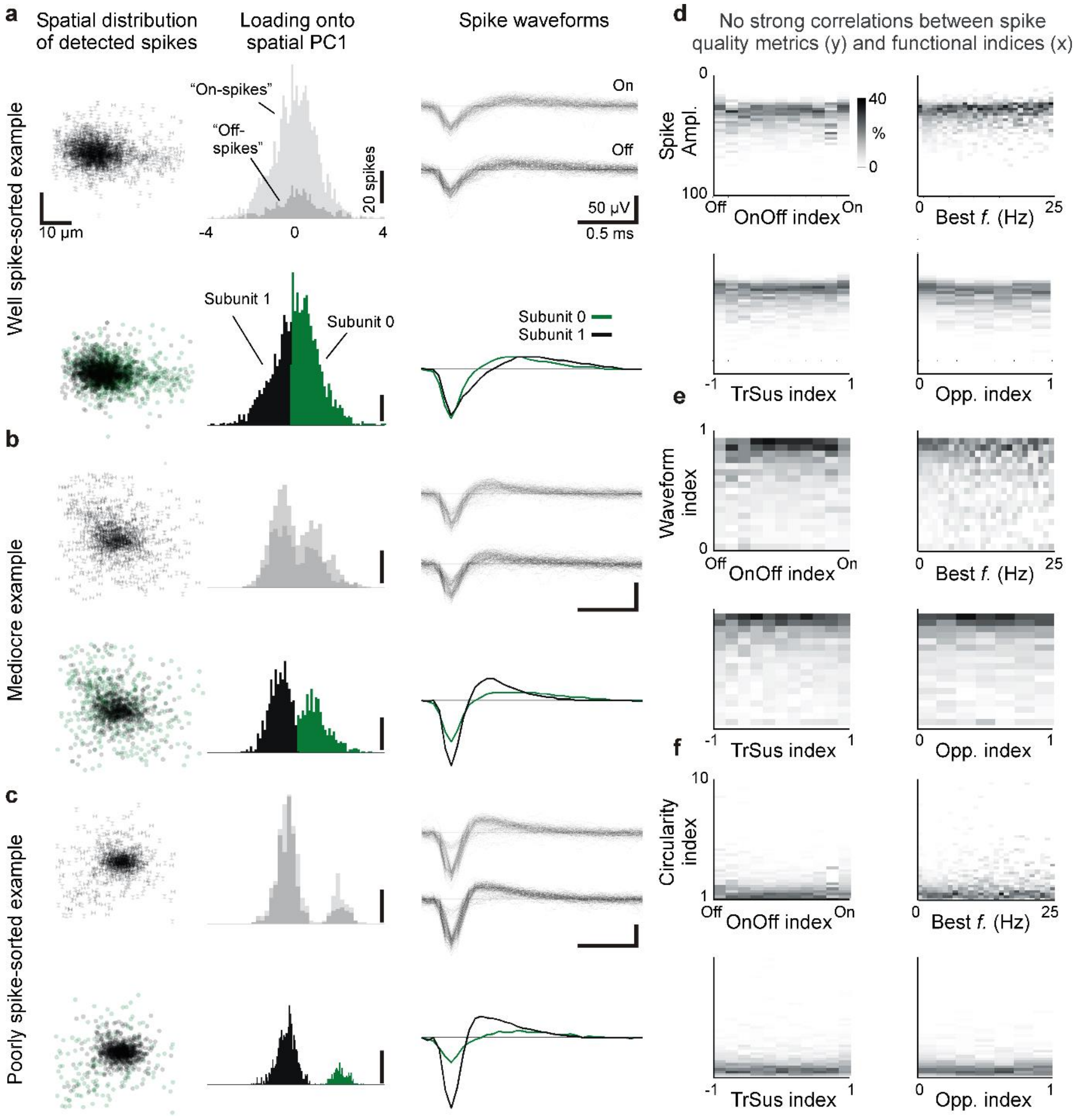

Supplemental Figure S2 – related to Figure 1. a-c, Examples of three spike-sorted units and their sorting-quality metrics, illustrating a well-sorted unit (a), a mediocre unit (b), and the worst unit in the dataset (c). Shown for each unit are the location of all detected "On and Off-spikes" in space (top left; see Methods for details), a histogram summarising waveform similarities of On- and Off-spikes based on principal component analysis (top middle), and all On- and Off-associated spike waveforms superimposed as indicated (top, right), and corresponding metrics for spikes artificially divided based on waveform similarities as shown (subunits 1 and 0 as indicated). Note how for the cell shown in (a), neither the artificial On- versus Off- division (top row) nor

the artificial division based on waveform shape (bottom row) lead to different distributions in the remaining parameters. By contrast, units shown in (b) and (c) show some bimodality in PC space (indicating that two units might have been merged during spike sorting). Note also, however, how none of these examples shows bimodality in On- versus Off-behaviour (which would indicate that an On cell had been merged with an Off cell to produce an OnOff cell). **d-f**, 2D histograms relating spike-sorting metrics (y axes) with functional indices used throughout the study (x-axis). Note that no 2D histogram shows an obvious diagonal, which would indicate a strong correlation between spike-sorting quality and inferred neuronal function.

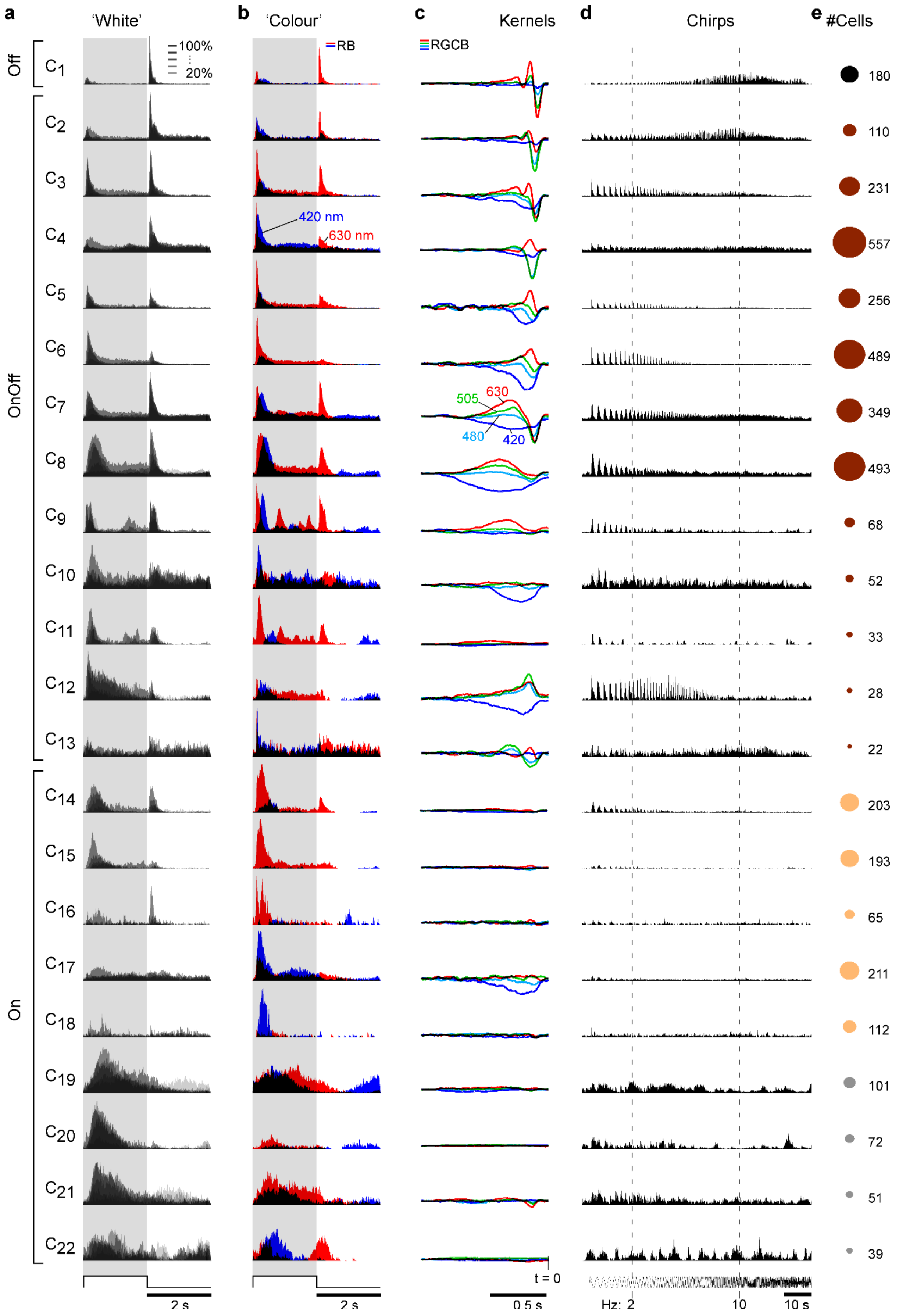

a
'White'
b
'Colour'
c
Kernels
d
Chirps
e
#Cells
100%
20%
RB
RGCB
Off
OnOff
On
C1
C2
C3
C4
C5
C6
C7
C8
C9
C10
C11
C12
C13
C14
C15
C16
C17
C18
C19
C20
C21
C22
420 nm
630 nm
505
630
480
420
180
110
231
557
256
489
349
493
68
52
33
28
22
203
193
65
211
112
101
72
51
39
2 s
2 s
t = 0
0.5 s
Hz: 2
10
10 s

Supplemental Figure S3 – related to Figures 2 and 3. a-e, Overview of each cluster's mean light responses to the presented battery of visual stimuli, showing every second 'white' contrast step (a), the 'red' and 'blue' colour steps (b), all four spectral kernels (c), chirp-responses (d) and an indication of the relative number of cells in each cluster (e). Full detail can be interactively explored in the online plotter at http://chicken-data.retinal-functomics.net/. Spike histograms are shown as peak-normalised to the largest response in each stimulus group.

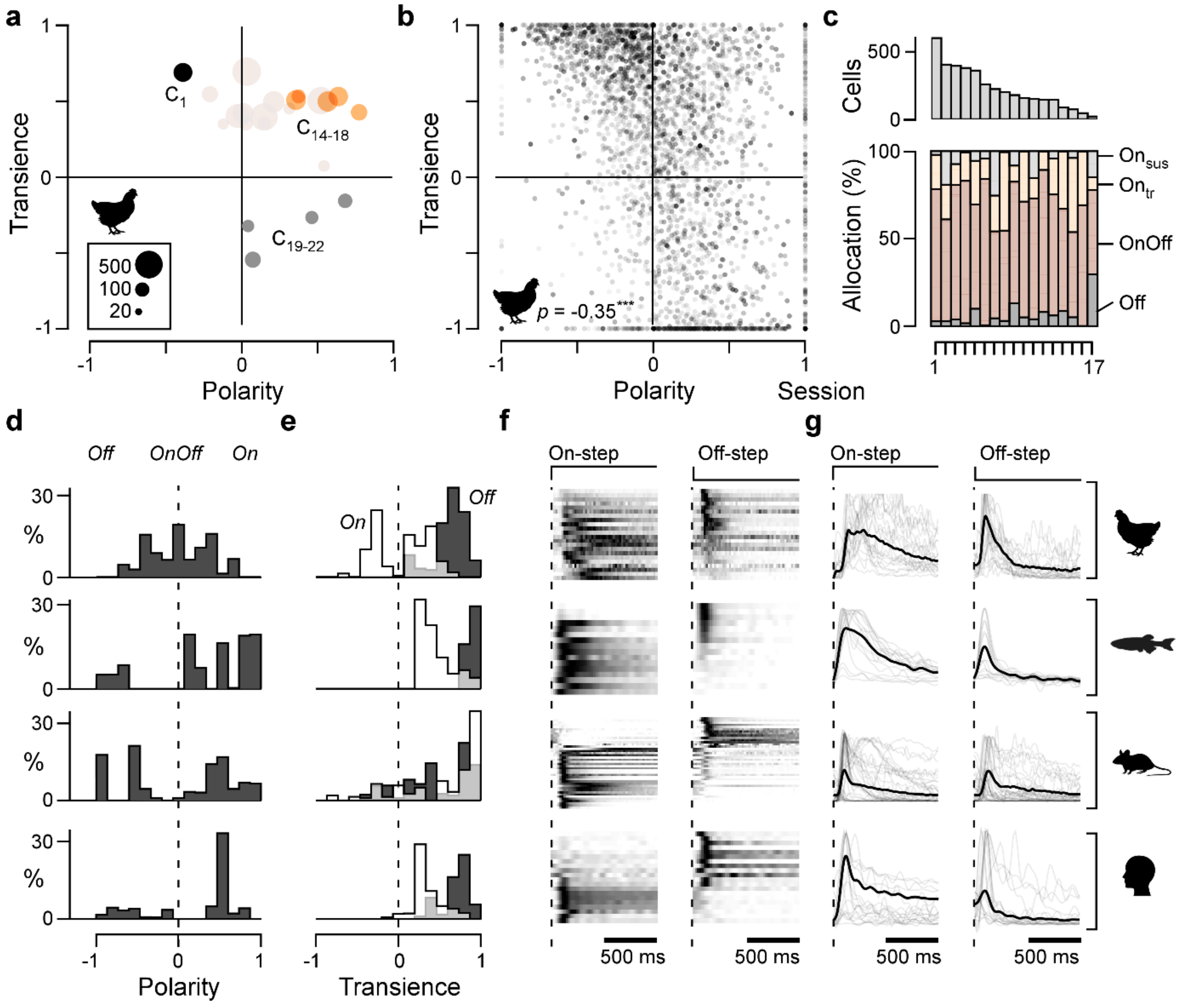

Supplemental Figure S4 – related to Figure 3. a, As Figure 3a, but based on each clusters' best responding CS, rather than the 100% contrast WS. This additional analysis was motivated by the weak responses to WS in several On-clusters. To highlight the kinetic differences among On-clusters, OnOff clusters $C_{2\text{-}13}$ are only plotted faintly into the background. **b**, as (a), but shown for each individual cell, correlation coefficient -0.35, two-sided correlation test p<0.001. **c**, Overview of group

and cluster allocations of all cells stemming from the 17 recording sessions. The colour coding indicates groups, while the more faintly plotted subdivisions therein refer to the individual clusters. **d-g**, comparison of polarity (d), transience of On- and Off-components (e) and corresponding RGC-type/cluster means (f,g) to a step of light (Methods), for (from top): chicken, larval zebrafish, mouse, and human. Faint lines in (g) indicate each type/cluster's response to an On- and Off-step (f), normalised to their respective peaks. The thick black lines indicate their mean. Note that for chicken and zebrafish, but not for mouse and human, On-responses are systematically broader compared to Off. Human silhouette in (g) from silhouettegarden.com.

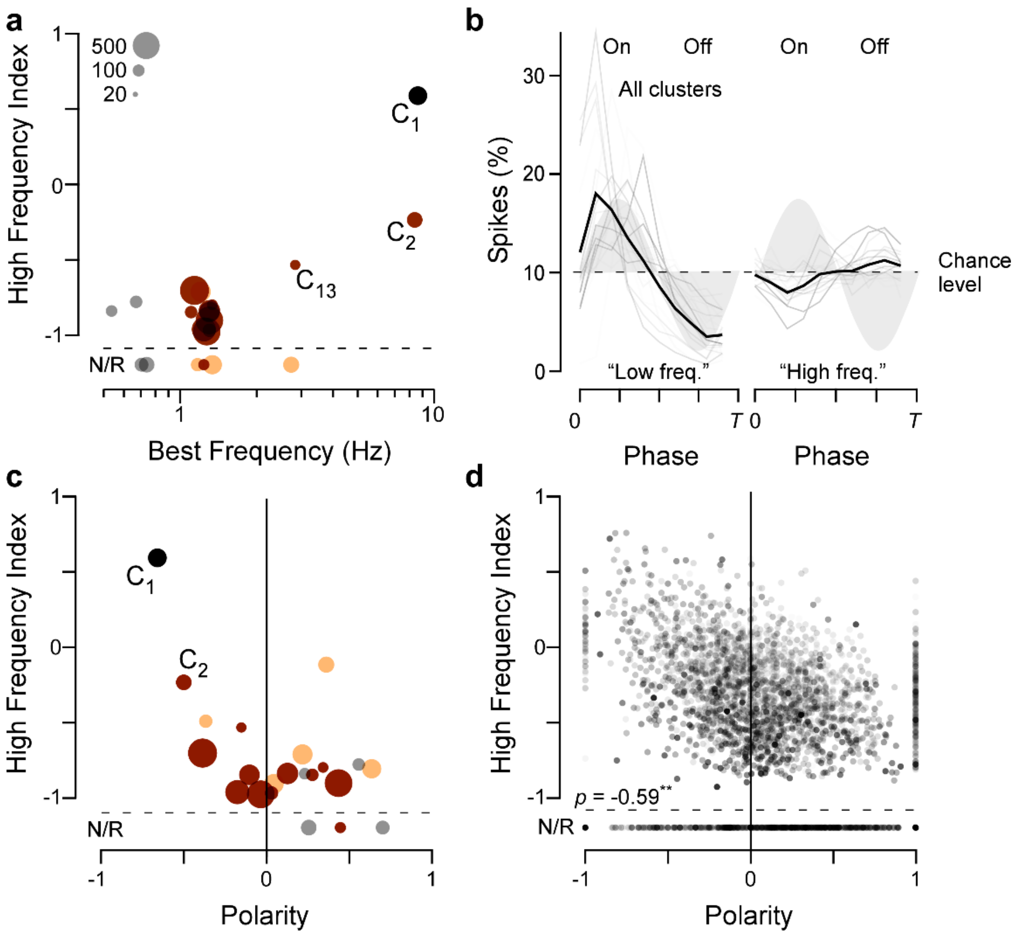

Supplemental Figure S5 – related to Figure 4. a, Relationship between each cluster's high-frequency index and best frequency (Methods) based on mean chirp responses. **b**, As Figure 4h, but here shown for all clusters (grey lines), and their mean (black). **c,d**, Relationship of high frequency index (Methods) and polarity, shown for

cluster means (c) and for all cells (d, Two-sided correlation test p = 0.0038, coefficient rho = -0.59).

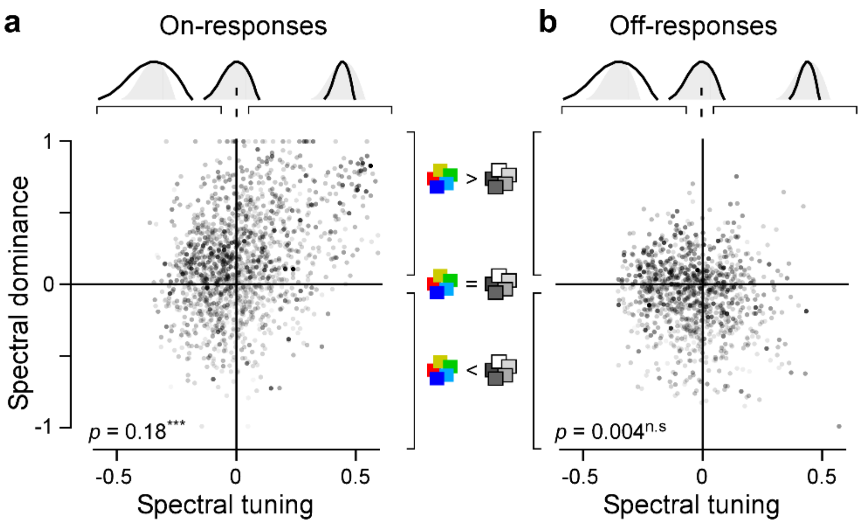

Supplemental Figure 6 – related to Figure 5. a,b, as Figure 5j,k, respectively, here shown for each cell. Two-sided correlation tests, for On and Off, respectively: $p < 0.001$ and 0.76; coefficient rho = 0.18 and 0.004.
