# Supplementary material for: Birds multiplex spectral and temporal visual information via retinal On- and Off-channels
Source: Nat Commun. 2023 Aug 31;14:5308. doi: 10.1038/s41467-023-41032-z (PMC10471707; doi:10.1038/s41467-023-41032-z)
Supplement: Supplementary file 3 — Description of Additional Supplementary Files [file 41467_2023_41032_MOESM3_ESM.pdf]

<u>Description of Additional Supplementary Files</u>

Supplementary Video S1: Related to Figure 1 | Example of electrical imaging of three retinal ganglion cells as shown in Figure 1.
