# Supplementary figures and images for: Birds multiplex spectral and temporal visual information via retinal On- and Off-channels
Source: Nat Commun. 2023 Aug 31;14:5308. doi: 10.1038/s41467-023-41032-z (PMC10471707; doi:10.1038/s41467-023-41032-z)

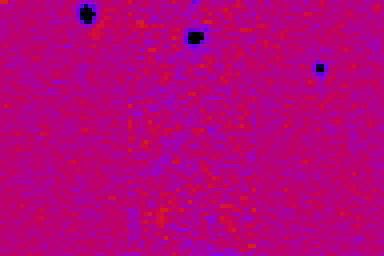

Supplement: Supplementary file 4 — Supplementary Video 1 [file 41467_2023_41032_MOESM4_ESM.gif]

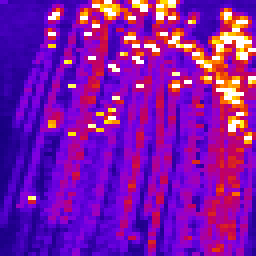

Supplement: Supplementary file 6 — Source Data [file 41467_2023_41032_MOESM6_ESM.zip › Fig 1j.png]
