# Supplementary material for: Birds multiplex spectral and temporal visual information via retinal On- and Off-channels
Source: Nat Commun. 2023 Aug 31;14:5308. doi: 10.1038/s41467-023-41032-z (PMC10471707; doi:10.1038/s41467-023-41032-z)
Supplement: Supplementary file 5 — Reporting Summary [file 41467_2023_41032_MOESM5_ESM.pdf]

nature portfolio

Corresponding author(s): Tom Baden

Last updated by author(s): 2023 07 13

Reporting Summary

Nature Portfolio wishes to improve the reproducibility of the work that we publish. This form provides structure for consistency and transparency in reporting. For further information on Nature Portfolio policies, see our Editorial Policies and the Editorial Policy Checklist.

Statistics

For all statistical analyses, confirm that the following items are present in the figure legend, table legend, main text, or Methods section.

n/a	Confirmed	
☐	☒	The exact sample size (*n*) for each experimental group/condition, given as a discrete number and unit of measurement
☒	☐	A statement on whether measurements were taken from distinct samples or whether the same sample was measured repeatedly
☐	☒	The statistical test(s) used AND whether they are one- or two-sided<br>*Only common tests should be described solely by name; describe more complex techniques in the Methods section.*
☒	☐	A description of all covariates tested
☒	☐	A description of any assumptions or corrections, such as tests of normality and adjustment for multiple comparisons
☐	☒	A full description of the statistical parameters including central tendency (e.g. means) or other basic estimates (e.g. regression coefficient) AND variation (e.g. standard deviation) or associated estimates of uncertainty (e.g. confidence intervals)
☐	☒	For null hypothesis testing, the test statistic (e.g. *F*, *t*, *r*) with confidence intervals, effect sizes, degrees of freedom and *P* value noted<br>*Give P values as exact values whenever suitable.*
☒	☐	For Bayesian analysis, information on the choice of priors and Markov chain Monte Carlo settings
☒	☐	For hierarchical and complex designs, identification of the appropriate level for tests and full reporting of outcomes
☐	☒	Estimates of effect sizes (e.g. Cohen's *d*, Pearson's *r*), indicating how they were calculated

Our web collection on statistics for biologists contains articles on many of the points above.

Software and code

Policy information about availability of computer code

Data collection

MEA recordings were performed on a BIOCAM X platform produced by 3Brain AG, Wädenswil, Switzerland. The chip was Arena HD-MEA. The software for data acquisition was Brainwave 4. It was run on a bespoke PC provided as part of the MEA by 3Brain. The integration time was set to medium, resulting in a sampling frequency of ~18 kHz.
Fiji version = 2.14.0
Brainwave 4 version = 4.5
Scripts are available on https://github.com/BadenLab/Nature_Comms_Paper_23.git

Python packages
aiofiles==22.1.0 ; python_version >= "3.10" and python_version < "4.0"
aiohttp==3.8.4 ; python_version >= "3.10" and python_version < "4.0"
aiosignal==1.3.1 ; python_version >= "3.10" and python_version < "4.0"
aiosqlite==0.18.0 ; python_version >= "3.10" and python_version < "4.0"
ansi2html==1.8.0 ; python_version >= "3.10" and python_version < "4.0"
anyio==3.6.2 ; python_version >= "3.10" and python_version < "4.0"
appnope==0.1.3 ; python_version >= "3.10" and python_version < "4.0" and sys_platform == "darwin" or python_version >= "3.10" and python_version < "4.0" and platform_system == "Darwin"
argon2-cffi-bindings==21.2.0 ; python_version >= "3.10" and python_version < "4.0"
argon2-cffi==21.3.0 ; python_version >= "3.10" and python_version < "4.0"
arrow==1.2.3 ; python_version >= "3.10" and python_version < "4.0"

```
astroid==2.14.2 ; python_version >= "3.10" and python_version < "4.0"
asttokens==2.2.1 ; python_version >= "3.10" and python_version < "4.0"
async-timeout==4.0.2 ; python_version >= "3.10" and python_version < "4.0"
attrs==22.2.0 ; python_version >= "3.10" and python_version < "4.0"
autopep8==1.6.0 ; python_version >= "3.10" and python_version < "4.0"
babel==2.11.0 ; python_version >= "3.10" and python_version < "4.0"
backcall==0.2.0 ; python_version >= "3.10" and python_version < "4.0"
beautifulsoup4==4.11.2 ; python_version >= "3.10" and python_version < "4.0"
bitarray==2.7.3 ; python_version >= "3.10" and python_version < "4.0"
black==23.1.0 ; python_version >= "3.10" and python_version < "4.0"
bleach==6.0.0 ; python_version >= "3.10" and python_version < "4.0"
blosc==1.11.1 ; python_version >= "3.10" and python_version < "4.0"
bokeh==2.4.3 ; python_version >= "3.10" and python_version < "4.0"
brotli==1.0.9 ; python_version >= "3.10" and python_version < "4.0"
caerus==0.1.9 ; python_version >= "3.10" and python_version < "4.0"
certifi==2022.12.7 ; python_version >= "3.10" and python_version < "4"
cffi==1.15.1 ; python_version >= "3.10" and python_version < "4.0"
charset-normalizer==3.0.1 ; python_version >= "3.10" and python_version < "4"
click==8.1.3 ; python_version >= "3.10" and python_version < "4.0"
cloudpickle==2.2.1 ; python_version >= "3.10" and python_version < "4.0"
colorama==0.4.6 ; python_version >= "3.10" and python_version < "4.0"
colorcet==3.0.1 ; python_version >= "3.10" and python_version < "4.0"
colorlover==0.3.0 ; python_version >= "3.10" and python_version < "4.0"
comm==0.1.2 ; python_version >= "3.10" and python_version < "4.0"
contourpy==1.0.7 ; python_version >= "3.10" and python_version < "4.0"
copier==8.0.0 ; python_version >= "3.10" and python_version < "4.0"
cramjam==2.6.2 ; python_version >= "3.10" and python_version < "4.0"
cycler==0.11.0 ; python_version >= "3.10" and python_version < "4.0"
cython==0.29.33 ; python_version >= "3.10" and python_version < "4.0"
dash-core-components==2.0.0 ; python_version >= "3.10" and python_version < "4.0"
dash-html-components==2.0.0 ; python_version >= "3.10" and python_version < "4.0"
dash-table==5.0.0 ; python_version >= "3.10" and python_version < "4.0"
dash==2.9.1 ; python_version >= "3.10" and python_version < "4.0"
dask==2023.3.1 ; python_version >= "3.10" and python_version < "4.0"
dataclasses-json==0.5.7 ; python_version >= "3.10" and python_version < "4.0"
datashader==0.14.4 ; python_version >= "3.10" and python_version < "4.0"
datashape==0.5.2 ; python_version >= "3.10" and python_version < "4.0"
debugpy==1.6.6 ; python_version >= "3.10" and python_version < "4.0"
decorator==5.1.1 ; python_version >= "3.10" and python_version < "4.0"
defusedxml==0.7.1 ; python_version >= "3.10" and python_version < "4.0"
dill==0.3.6 ; python_version >= "3.10" and python_version < "4.0"
diptest==0.5.2 ; python_version >= "3.10" and python_version < "4.0"
docstring-to-markdown==0.11 ; python_version >= "3.10" and python_version < "4.0"
dunamai==1.17.0 ; python_version >= "3.10" and python_version < "4.0"
entrypoints==0.4 ; python_version >= "3.10" and python_version < "4.0"
executing==1.2.0 ; python_version >= "3.10" and python_version < "4.0"
fastjsonschema==2.16.2 ; python_version >= "3.10" and python_version < "4.0"
fastparquet==2023.2.0 ; python_version >= "3.10" and python_version < "4.0"
findpeaks==2.4.7 ; python_version >= "3.10" and python_version < "4.0"
flake8==6.0.0 ; python_version >= "3.10" and python_version < "4.0"
flask==2.2.3 ; python_version >= "3.10" and python_version < "4.0"
fonttools==4.38.0 ; python_version >= "3.10" and python_version < "4.0"
fqdn==1.5.1 ; python_version >= "3.10" and python_version < "4"
frozenlist==1.3.3 ; python_version >= "3.10" and python_version < "4.0"
fsspec==2023.3.0 ; python_version >= "3.10" and python_version < "4.0"
funcy==2.0 ; python_version >= "3.10" and python_version < "4.0"
gorillacompression==0.2.1 ; python_version >= "3.10" and python_version < "4.0"
greenlet==2.0.2 ; python_version >= "3.10" and (platform_machine == "aarch64" or platform_machine == "ppc64le" or platform_machine ==
"x86_64" or platform_machine == "amd64" or platform_machine == "AMD64" or platform_machine == "win32" or platform_machine ==
"WIN32") and python_version < "4.0"
h5py==3.8.0 ; python_version >= "3.10" and python_version < "4.0"
herdingspikes==0.3.102 ; python_version >= "3.10" and python_version < "4.0"
holoviews==1.15.4 ; python_version >= "3.10" and python_version < "4.0"
hvplot==0.8.3 ; python_version >= "3.10" and python_version < "4.0"
idna==3.4 ; python_version >= "3.10" and python_version < "4"
imageio==2.31.0 ; python_version >= "3.10" and python_version < "4.0"
importlib-metadata==5.2.0 ; python_version >= "3.10" and python_version < "4.0"
ipykernel==6.21.2 ; python_version >= "3.10" and python_version < "4.0"
ipympl==0.9.2 ; python_version >= "3.10" and python_version < "4.0"
```

```
ipython-genutils==0.2.0 ; python_version >= "3.10" and python_version < "4.0"
ipython==8.10.0 ; python_version >= "3.10" and python_version < "4.0"
ipywidgets==7.7.2 ; python_version >= "3.10" and python_version < "4.0"
isoduration==20.11.0 ; python_version >= "3.10" and python_version < "4.0"
isort==5.12.0 ; python_version >= "3.10" and python_version < "4.0"
itsdangerous==2.1.2 ; python_version >= "3.10" and python_version < "4.0"
jedi==0.18.2 ; python_version >= "3.10" and python_version < "4.0"
jinja2-ansible-filters==1.3.2 ; python_version >= "3.10" and python_version < "4.0"
jinja2==3.1.2 ; python_version >= "3.10" and python_version < "4.0"
joblib==1.2.0 ; python_version >= "3.10" and python_version < "4.0"
json5==0.9.11 ; python_version >= "3.10" and python_version < "4.0"
jsonpointer==2.3 ; python_version >= "3.10" and python_version < "4.0"
jsonschema==4.17.3 ; python_version >= "3.10" and python_version < "4.0"
jsonschema[format-nongpl]==4.17.3 ; python_version >= "3.10" and python_version < "4.0"
jupyter-ai-magics==0.5.0 ; python_version >= "3.10" and python_version < "4.0"
jupyter-ai==0.5.0 ; python_version >= "3.10" and python_version < "4.0"
jupyter-black==0.3.3 ; python_version >= "3.10" and python_version < "4.0"
jupyter-client==8.0.2 ; python_version >= "3.10" and python_version < "4.0"
jupyter-console==6.6.3 ; python_version >= "3.10" and python_version < "4.0"
jupyter-core==5.2.0 ; python_version >= "3.10" and python_version < "4.0"
jupyter-dash==0.4.2 ; python_version >= "3.10" and python_version < "4.0"
jupyter-events==0.5.0 ; python_version >= "3.10" and python_version < "4.0"
jupyter-lsp==1.5.1 ; python_version >= "3.10" and python_version < "4.0"
jupyter-server-fileid==0.6.0 ; python_version >= "3.10" and python_version < "4.0"
jupyter-server-terminals==0.4.4 ; python_version >= "3.10" and python_version < "4.0"
jupyter-server-ydoc==0.6.1 ; python_version >= "3.10" and python_version < "4.0"
jupyter-server==2.2.1 ; python_version >= "3.10" and python_version < "4.0"
jupyter-ydoc==0.2.2 ; python_version >= "3.10" and python_version < "4.0"
jupyter==1.0.0 ; python_version >= "3.10" and python_version < "4.0"
jupyterblack==0.4.0 ; python_version >= "3.10" and python_version < "4.0"
jupyterlab-lsp==3.10.2 ; python_version >= "3.10" and python_version < "4.0"
jupyterlab-pygments==0.2.2 ; python_version >= "3.10" and python_version < "4.0"
jupyterlab-server==2.19.0 ; python_version >= "3.10" and python_version < "4.0"
jupyterlab-widgets==1.1.2 ; python_version >= "3.10" and python_version < "4.0"
jupyterlab==3.6.1 ; python_version >= "3.10" and python_version < "4.0"
kaleido==0.1.0.post1 ; python_version >= "3.10" and python_version < "4.0"
kiwisolver==1.4.4 ; python_version >= "3.10" and python_version < "4.0"
langchain==0.0.144 ; python_version >= "3.10" and python_version < "4.0"
lazy-loader==0.2 ; python_version >= "3.10" and python_version < "4.0"
lazy-object-proxy==1.9.0 ; python_version >= "3.10" and python_version < "4.0"
llvmlite==0.39.1 ; python_version >= "3.10" and python_version < "4.0"
locket==1.0.0 ; python_version >= "3.10" and python_version < "4.0"
markdown==3.4.3 ; python_version >= "3.10" and python_version < "4.0"
markupsafe==2.1.2 ; python_version >= "3.10" and python_version < "4.0"
marshmallow-enum==1.5.1 ; python_version >= "3.10" and python_version < "4.0"
marshmallow==3.19.0 ; python_version >= "3.10" and python_version < "4.0"
matplotlib-inline==0.1.6 ; python_version >= "3.10" and python_version < "4.0"
matplotlib==3.7.0 ; python_version >= "3.10" and python_version < "4.0"
mccabe==0.7.0 ; python_version >= "3.10" and python_version < "4.0"
memory-profiler==0.61.0 ; python_version >= "3.10" and python_version < "4.0"
mistune==2.0.5 ; python_version >= "3.10" and python_version < "4.0"
modin-spreadsheet==0.1.2 ; python_version >= "3.10" and python_version < "4.0"
modin==0.20.0 ; python_version >= "3.10" and python_version < "4.0"
mpi4py==3.1.4 ; python_version >= "3.10" and python_version < "4.0"
mpmath==1.2.1 ; python_version >= "3.10" and python_version < "4.0"
multidict==6.0.4 ; python_version >= "3.10" and python_version < "4.0"
multipledispatch==0.6.0 ; python_version >= "3.10" and python_version < "4.0"
mypy-extensions==1.0.0 ; python_version >= "3.10" and python_version < "4.0"
nbclassic==0.5.1 ; python_version >= "3.10" and python_version < "4.0"
nbclient==0.7.2 ; python_version >= "3.10" and python_version < "4.0"
nbconvert==7.2.9 ; python_version >= "3.10" and python_version < "4.0"
nbformat==5.7.3 ; python_version >= "3.10" and python_version < "4.0"
neo==0.11.1 ; python_version >= "3.10" and python_version < "4.0"
nest-asyncio==1.5.6 ; python_version >= "3.10" and python_version < "4.0"
networkx==3.0 ; python_version >= "3.10" and python_version < "4.0"
nodeenv==1.7.0 ; python_version >= "3.10" and python_version < "4.0"
notebook-shim==0.2.2 ; python_version >= "3.10" and python_version < "4.0"
notebook==6.5.2 ; python_version >= "3.10" and python_version < "4.0"
```

```
numba==0.56.4 ; python_version >= "3.10" and python_version < "4.0"
numexpr==2.8.4 ; python_version >= "3.10" and python_version < "4.0"
numpy==1.23.2 ; python_version >= "3.10" and python_version < "4.0"
openai==0.27.6 ; python_version >= "3.10" and python_version < "4.0"
openapi-schema-pydantic==1.2.4 ; python_version >= "3.10" and python_version < "4.0"
opencv-python==4.7.0.72 ; python_version >= "3.10" and python_version < "4.0"
packaging==23.0 ; python_version >= "3.10" and python_version < "4.0"
pandas==1.5.3 ; python_version >= "3.10" and python_version < "4.0"
pandocfilters==1.5.0 ; python_version >= "3.10" and python_version < "4.0"
panel==0.14.4 ; python_version >= "3.10" and python_version < "4.0"
param==1.13.0 ; python_version >= "3.10" and python_version < "4.0"
parso==0.8.3 ; python_version >= "3.10" and python_version < "4.0"
partd==1.3.0 ; python_version >= "3.10" and python_version < "4.0"
pathspec==0.11.0 ; python_version >= "3.10" and python_version < "4.0"
patsy==0.5.3 ; python_version >= "3.10" and python_version < "4.0"
peakdetect==1.1 ; python_version >= "3.10" and python_version < "4.0"
pexpect==4.8.0 ; python_version >= "3.10" and python_version < "4.0" and sys_platform != "win32"
physt==0.5.3 ; python_version >= "3.10" and python_version < "4.0"
pickleshare==0.7.5 ; python_version >= "3.10" and python_version < "4.0"
pillow==9.4.0 ; python_version >= "3.10" and python_version < "4.0"
platformdirs==3.0.0 ; python_version >= "3.10" and python_version < "4.0"
plotly==5.13.0 ; python_version >= "3.10" and python_version < "4.0"
pluggy==1.0.0 ; python_version >= "3.10" and python_version < "4.0"
plumbum==1.8.2 ; python_version >= "3.10" and python_version < "4.0"
probeinterface==0.2.16 ; python_version >= "3.10" and python_version < "4.0"
prometheus-client==0.16.0 ; python_version >= "3.10" and python_version < "4.0"
prompt-toolkit==3.0.36 ; python_version >= "3.10" and python_version < "4.0"
psutil==5.9.4 ; python_version >= "3.10" and python_version < "4.0"
ptyprocess==0.7.0 ; python_version >= "3.10" and python_version < "4.0" and sys_platform != "win32" or python_version >= "3.10" and
python_version < "4.0" and os_name != "nt"
pure-eval==0.2.2 ; python_version >= "3.10" and python_version < "4.0"
pycodestyle==2.10.0 ; python_version >= "3.10" and python_version < "4.0"
pycparser==2.21 ; python_version >= "3.10" and python_version < "4.0"
pyct==0.5.0 ; python_version >= "3.10" and python_version < "4.0"
pydantic==1.10.7 ; python_version >= "3.10" and python_version < "4.0"
pydocstyle==6.2.3 ; python_version >= "3.10" and python_version < "4.0"
pyflakes==3.0.1 ; python_version >= "3.10" and python_version < "4.0"
pygments==2.14.0 ; python_version >= "3.10" and python_version < "4.0"
pylint==2.16.2 ; python_version >= "3.10" and python_version < "4.0"
pyparsing==3.0.9 ; python_version >= "3.10" and python_version < "4.0"
pyrsistent==0.19.3 ; python_version >= "3.10" and python_version < "4.0"
pyspike @ git+https://github.com/mariomulansky/PySpike.git@HEAD ; python_version >= "3.10" and python_version < "4.0"
python-dateutil==2.8.2 ; python_version >= "3.10" and python_version < "4.0"
python-json-logger==2.0.6 ; python_version >= "3.10" and python_version < "4.0"
python-lsp-jsonrpc==1.0.0 ; python_version >= "3.10" and python_version < "4.0"
python-lsp-server[all]==1.7.1 ; python_version >= "3.10" and python_version < "4.0"
pytoolconfig[global]==1.2.5 ; python_version >= "3.10" and python_version < "4.0"
pytz==2022.7.1 ; python_version >= "3.10" and python_version < "4.0"
pyviz-comms==2.2.1 ; python_version >= "3.10" and python_version < "4.0"
pywavelets==1.4.1 ; python_version >= "3.10" and python_version < "4.0"
pywin32==305 ; sys_platform == "win32" and platform_python_implementation != "PyPy" and python_version >= "3.10" and python_version
< "4.0" or platform_system == "Windows" and platform_python_implementation != "PyPy" and python_version >= "3.10" and python_version
< "4.0"
pywinpty==2.0.10 ; python_version >= "3.10" and python_version < "4.0" and os_name == "nt"
pyyaml-include==1.3.1 ; python_version >= "3.10" and python_version < "4.0"
pyyaml==6.0 ; python_version >= "3.10" and python_version < "4.0"
pyzmq==25.0.0 ; python_version >= "3.10" and python_version < "4.0"
qgrid==1.3.1 ; python_version >= "3.10" and python_version < "4.0"
qtconsole==5.4.2 ; python_version >= "3.10" and python_version < "4.0"
qtpy==2.3.1 ; python_version >= "3.10" and python_version < "4.0"
quantities==0.14.0 ; python_version >= "3.10" and python_version < "4.0"
questionary==1.10.0 ; python_version >= "3.10" and python_version < "4.0"
requests==2.28.2 ; python_version >= "3.10" and python_version < "4"
retrying==1.3.4 ; python_version >= "3.10" and python_version < "4.0"
rfc3339-validator==0.1.4 ; python_version >= "3.10" and python_version < "4.0"
rfc3986-validator==0.1.1 ; python_version >= "3.10" and python_version < "4.0"
rope==1.7.0 ; python_version >= "3.10" and python_version < "4.0"
safer==4.4.1 ; python_version >= "3.10" and python_version < "4.0"
scikit-image==0.21.0 ; python_version >= "3.10" and python_version < "4.0"
```

```
scikit-learn==1.2.1 ; python_version >= "3.10" and python_version < "4.0"
scipy==1.9.3 ; python_version >= "3.10" and python_version < "4.0"
send2trash==1.8.0 ; python_version >= "3.10" and python_version < "4.0"
setuptools==67.3.1 ; python_version >= "3.10" and python_version < "4.0"
six==1.16.0 ; python_version >= "3.10" and python_version < "4.0"
sniffio==1.3.0 ; python_version >= "3.10" and python_version < "4.0"
snowballstemmer==2.2.0 ; python_version >= "3.10" and python_version < "4.0"
soupsieve==2.4 ; python_version >= "3.10" and python_version < "4.0"
spikeinterface==0.97.0 ; python_version >= "3.10" and python_version < "4.0"
spyking-circus==1.1.0 ; python_version >= "3.10" and python_version < "4.0"
sqlalchemy==1.4.47 ; python_version >= "3.10" and python_version < "4.0"
stack-data==0.6.2 ; python_version >= "3.10" and python_version < "4.0"
statsmodels==0.13.5 ; python_version >= "3.10" and python_version < "4.0"
sympy==1.11.1 ; python_version >= "3.10" and python_version < "4.0"
tenacity==8.2.1 ; python_version >= "3.10" and python_version < "4.0"
terminado==0.17.1 ; python_version >= "3.10" and python_version < "4.0"
threadpoolctl==3.1.0 ; python_version >= "3.10" and python_version < "4.0"
tifffile==2023.4.12 ; python_version >= "3.10" and python_version < "4.0"
tinycss2==1.2.1 ; python_version >= "3.10" and python_version < "4.0"
tokenize-rt==5.0.0 ; python_version >= "3.10" and python_version < "4.0"
toml==0.10.2 ; python_version >= "3.10" and python_version < "4.0"
tomli==2.0.1 ; python_version >= "3.10" and python_version < "3.11"
tomlkit==0.11.6 ; python_version >= "3.10" and python_version < "4.0"
toolz==0.12.0 ; python_version >= "3.10" and python_version < "4.0"
tornado==6.2 ; python_version >= "3.10" and python_version < "4.0"
tqdm==4.64.1 ; python_version >= "3.10" and python_version < "4.0"
traitlets==5.9.0 ; python_version >= "3.10" and python_version < "4.0"
tslearn==0.5.3.2 ; python_version >= "3.10" and python_version < "4.0"
typing-extensions==4.5.0 ; python_version >= "3.10" and python_version < "4.0"
typing-inspect==0.8.0 ; python_version >= "3.10" and python_version < "4.0"
ujson==5.7.0 ; python_version >= "3.10" and python_version < "4.0"
uri-template==1.2.0 ; python_version >= "3.10" and python_version < "4.0"
urllib3==1.26.14 ; python_version >= "3.10" and python_version < "4"
wcwidth==0.2.6 ; python_version >= "3.10" and python_version < "4.0"
webcolors==1.12 ; python_version >= "3.10" and python_version < "4.0"
webencodings==0.5.1 ; python_version >= "3.10" and python_version < "4.0"
websocket-client==1.5.1 ; python_version >= "3.10" and python_version < "4.0"
werkzeug==2.2.3 ; python_version >= "3.10" and python_version < "4.0"
wget==3.2 ; python_version >= "3.10" and python_version < "4.0"
whatthepatch==1.0.4 ; python_version >= "3.10" and python_version < "4.0"
widgetsnbextension==3.6.2 ; python_version >= "3.10" and python_version < "4.0"
wrapt==1.14.1 ; python_version >= "3.10" and python_version < "4.0"
xarray==2023.3.0 ; python_version >= "3.10" and python_version < "4.0"
y-py==0.5.5 ; python_version >= "3.10" and python_version < "4.0"
yapf==0.32.0 ; python_version >= "3.10" and python_version < "4.0"
yarl==1.8.2 ; python_version >= "3.10" and python_version < "4.0"
ypy-websocket==0.8.2 ; python_version >= "3.10" and python_version < "4.0"
zipp==3.15.0 ; python_version >= "3.10" and python_version < "4.0"
zstandard==0.21.0 ; python_version >= "3.10" and python_version < "4.0"
```

Data analysis

Data analysis was performed using custom written Python (v. 3.9.5, Python Software Foundation, Wilmington, Delaware, USA) scripts executed in JuypterLab, IGOR Pro 6.3 (Wavemetrics), Fiji (NIH) and Matlab R2019b / R2020b (Mathworks).

For manuscripts utilizing custom algorithms or software that are central to the research but not yet described in published literature, software must be made available to editors and reviewers. We strongly encourage code deposition in a community repository (e.g. GitHub). See the Nature Portfolio guidelines for submitting code & software for further information.

Data

Policy information about availability of data

All manuscripts must include a data availability statement. This statement should provide the following information, where applicable:

- Accession codes, unique identifiers, or web links for publicly available datasets
- A description of any restrictions on data availability
- For clinical datasets or third party data, please ensure that the statement adheres to our policy

All pre-processed spike data (cellwise trial-averaged means and kernels) are available for interactive display and download from http://chicken-data.retinal-functomics.net/. The underlying individual spike timings, corresponding stimuli, and an explanation of the file structures can be downloaded from the same site.

Human research participants

Policy information about studies involving human research participants and Sex and Gender in Research.

Reporting on sex and gender	N/A
Population characteristics	N/A
Recruitment	N/A
Ethics oversight	N/A

Note that full information on the approval of the study protocol must also be provided in the manuscript.

Field-specific reporting

Please select the one below that is the best fit for your research. If you are not sure, read the appropriate sections before making your selection.

☒ Life sciences ☐ Behavioural & social sciences ☐ Ecological, evolutionary & environmental sciences

For a reference copy of the document with all sections, see nature.com/documents/nr-reporting-summary-flat.pdf

Life sciences study design

All studies must disclose on these points even when the disclosure is negative.

Sample size	No statistical methods were used to predetermine sample size. This study did not compare different groups experimentally. Effect size was determined by time availability and experimental success rate.
Data exclusions	None
Replication	We replicated recordings across 14 different animals with similar results. We outline the used protocol in detail in this study.
Randomization	This study did not have any experimental groups. Thus randomization wasn't relevant.
Blinding	Blinding was not relevant to this study, as no groups were experimentally compared.

Reporting for specific materials, systems and methods

We require information from authors about some types of materials, experimental systems and methods used in many studies. Here, indicate whether each material, system or method listed is relevant to your study. If you are not sure if a list item applies to your research, read the appropriate section before selecting a response.

Materials & experimental systems

n/a	Involved in the study	
☒	☐	Antibodies
☒	☐	Eukaryotic cell lines
☒	☐	Palaeontology and archaeology
☐	☒	Animals and other organisms
☒	☐	Clinical data
☒	☐	Dual use research of concern

Methods

n/a	Involved in the study	
☒	☐	ChIP-seq
☒	☐	Flow cytometry
☒	☐	MRI-based neuroimaging

Animals and other research organisms

Policy information about studies involving animals; ARRIVE guidelines recommended for reporting animal research, and Sex and Gender in Research

Laboratory animals	All procedures were performed in accordance with the UK Animals (Scientific Procedures) act 1986 and approved by the animal welfare committee of the University of Sussex. Male chicks, breed Shaver Brown, aged between 1 and 14-days post hatching were obtained from Joice and Hill (part of Hendrix Genetics, Peterborough, UK) and kept in a specifically designed cage in the university's animal facility. Food (chick crumb) was provided ad libitum and elements for facilitating play behaviour were provided. Chicks were

	kept on a 12h:12h light:dark cycle, and never kept in isolation for prolonged periods of time.
Wild animals	No wild animals were used in this study.
Reporting on sex	All experiments were performed in male gallus gallus. The study studied 14 male and 0 female chicken.
Field-collected samples	No field collected samples were used for this study.
Ethics oversight	UK Animals (Scientific Procedures) act 1986, approved by the animal welfare committee of the University of Sussex

Note that full information on the approval of the study protocol must also be provided in the manuscript.
