# Supplementary material for: Birds multiplex spectral and temporal visual information via retinal On- and Off-channels
Source: Nat Commun. 2023 Aug 31;14:5308. doi: 10.1038/s41467-023-41032-z (PMC10471707; doi:10.1038/s41467-023-41032-z)
Supplement: Supplementary file 6 — Source Data [file 41467_2023_41032_MOESM6_ESM.zip › Source Data information.docx]

All preprocessed spike data is available for interactive viewing and download and online viewing from

<https://chicken-dataset-plot.herokuapp.com/apps/overview_plot>

Any individual figure panels that are no deposited as part of the source data can be obtained from the online plotter (For example the cluster summary plots in Figure 4j-m).

The remaining data that leads to the panels in the figures are deposited individually.

Most are in the form of pxp files, which are “Packed IGRO Pro” files. They can be read by downloading Igor Pro from the Wavemetrics Website (for free, 1 month full functionality trial, and student licences are free): <https://www.wavemetrics.com/>

The remaining datasets are either as Python arrays or as Tiff files (which can be read, for example, by ImageJ)
